# Extensor Hallucis Longus Contracture Following Intramedullary Nailing for a Pilon Fracture

**DOI:** 10.7759/cureus.78195

**Published:** 2025-01-29

**Authors:** Nicholas J Tsavaris, Mitchell J Lomis, Sean Gordon, Parker Vaughan, Scarlett Schneider

**Affiliations:** 1 Orthopedic Surgery, Augusta University Medical College of Georgia, Athens, USA; 2 Sports Medicine, Augusta University/University of Georgia Medical Partnership, Athens, USA

**Keywords:** intramedullary nails, lower extremity trauma, orthopedic intervention, tendon contracture, tenolysis, tibial pilon fracture

## Abstract

Extensor hallucis longus (EHL) fibrosis is an uncommon complication following a pilon fracture. This is caused by compartment syndrome from fracture immobilization followed by adhesion of muscle or tendon. Only a few cases of EHL fibrosis secondary to a post-traumatic lower leg fracture have been reported. This paper reports on a rare case of isolated EHL tendon fibrosis after a pilon fracture. It will outline the treatment received and the current prognosis of the patient, while also discussing methods for prevention of this complication.

## Introduction

Pilon fractures of the tibia, also known as tibial plafond fractures, are a type of fracture that involves the distal tibia and ankle joint. More specifically, pilon fractures occur when a large axial force drives the talus into the distal tibia while the foot is in dorsiflexion causing comminuted distal fracture of the tibia, often with extensive soft tissue damage and fibular involvement. Pilon fractures are relatively rare, and account for less than 10% of lower leg fractures [1].

Tenofibrosis of the extensor hallucis longus (EHL) following a pilon fracture of the tibia treated with open reduction and internal fixation using an intramedullary (IM) nail represents a rare cause of isolated hallux extensor contracture [2]. Contracture is defined by a reduction in the range of motion (ROM) in response to passive or active movements and is commonly seen due to ischemia, myonecrosis, fibroblast proliferation, and tissue adhesion [3]. Additionally, contracture can result from immobilization due to injury or lack of exercise, spasticity secondary to a neurological condition, joint related pathologies like arthritis, or general muscle weakness [4,5].

While joint immobilization is crucial to the healing process of an injury, it complicates rehabilitation due to the risk of contracture from prolonged stasis [6]. Muscle and tendon tissue requires constant tension to maintain appropriate kinetic/mechanical capability [7]. This is why the use of mobility and light resistance methods to reduce the chance of this complication are crucial to the healing process [8]. In general, the more common causes of EHL tenofibrosis that have previously been reported include anterior compartment syndrome, direct injury, and entrapment or adhesion of the muscle or tendon [2].

Here, we present a case of isolated hallux extensor contracture due to tenofibrosis of the EHL following a right distal tibial pilon fracture with associated diaphyseal fibula fracture without evidence of compartment syndrome. At one-month follow-up after tenolysis of the right EHL tendon with platelet-rich plasma (PRP), the patient demonstrated weak active great toe dorsiflexion. The patient is continuing physical therapy to improve mobility and function. There have been a few cases of EHL fibrosis following a tibiofibular fracture reported in the literature [2,9,10,11]. However, this case is a rare example of this complication associated specifically with a pilon fracture. 

## Case presentation

**Figure 1:**
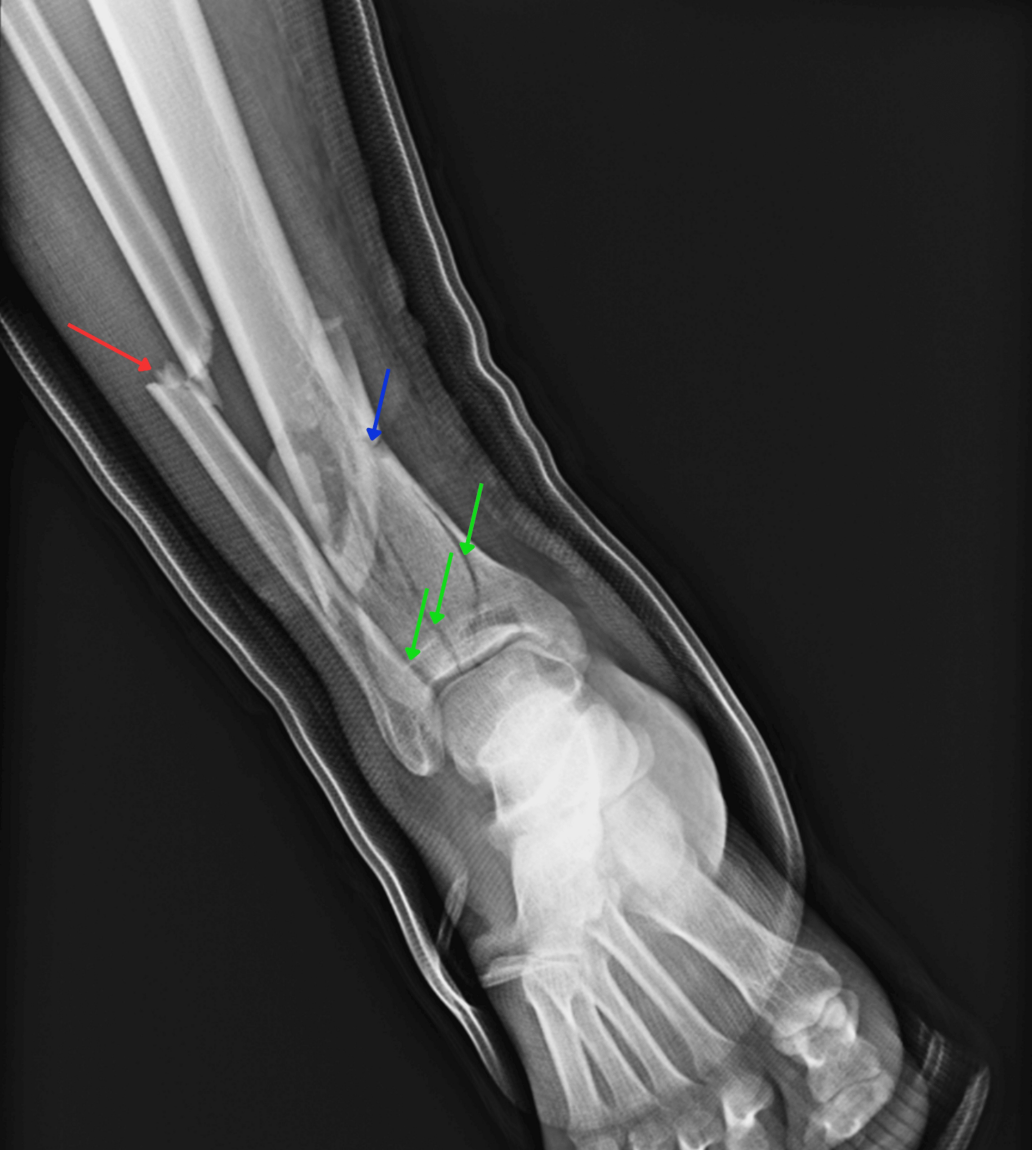
Anteroposterior film demonstrating original pilon fracture of left tibia with additional fibular involvement Fracture lines indicative of a pilon fracture are shown by the green arrows. Tibial and fibular shaft fractures are indicated by blue and red arrows, respectively.

A 56-year-old male presented to an orthopedic clinic with right leg pain following an accident two days prior in which his leg was crushed between two automobiles. He was initially diagnosed with a right ankle fracture and placed in a sugar tong splint. The patient was instructed to follow-up with orthopedics. He reported 10/10 constant sharp pain accompanied by a throbbing sensation and swelling in his right toes. X-ray film demonstrated a displaced, oblique fibular fracture with a significantly displaced and comminuted tibia fracture (Figure 1).

**Figure 2:**
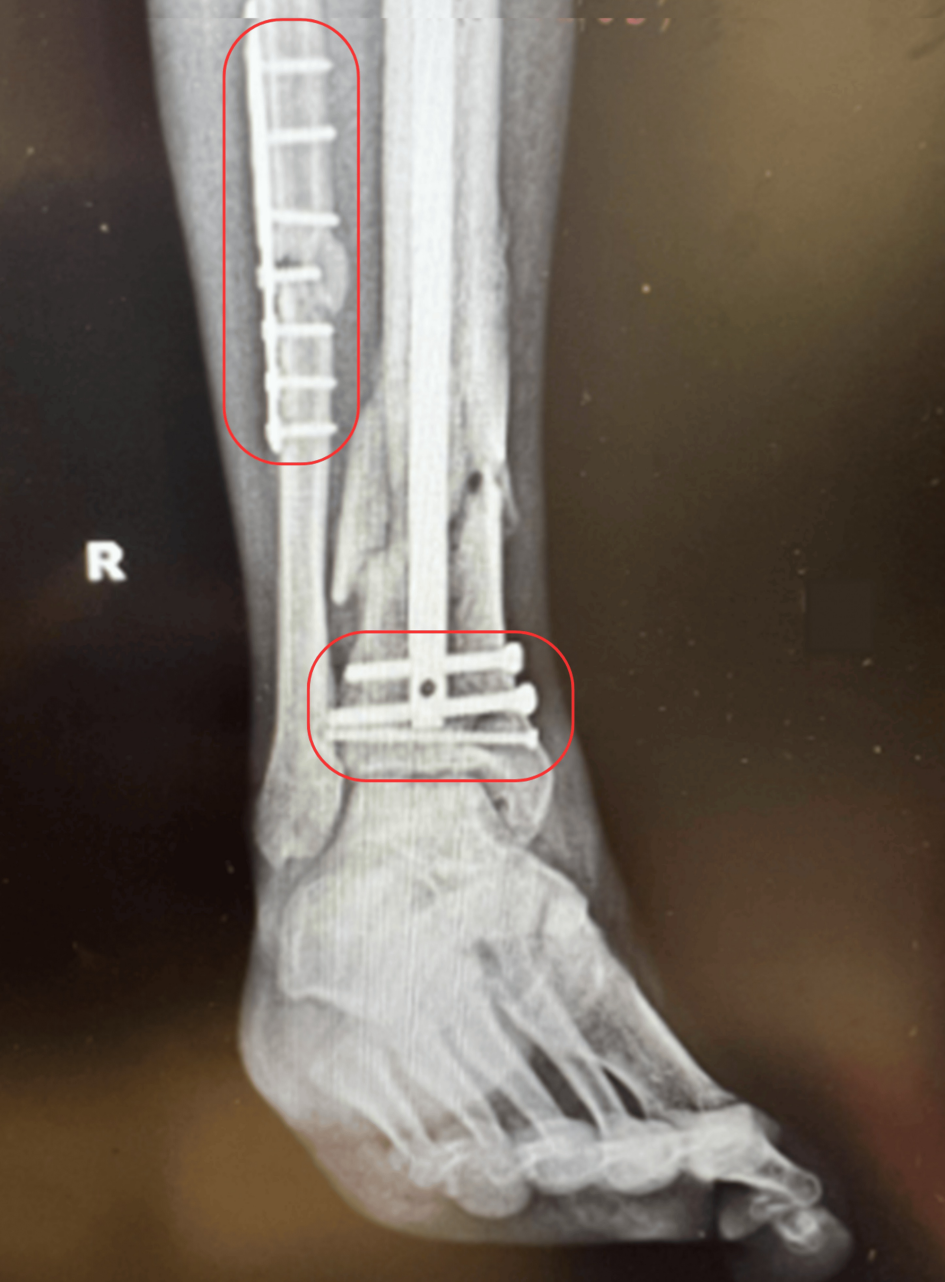
Mortise X-ray film after ORIF procedure Areas of fixation are indicated by red boxes showing good alinement post ORIF procedure. ORIF: Open reduction internal fixation

Open reduction internal fixation (ORIF) with an IM nailing of the tibia was recommended to the patient with risks and benefits discussed. The patient decided to proceed with surgery which was performed the subsequent week and was successful for proper placement of the hardware and appropriate reduction of the fracture shown below (Figure 2). The patient was given sequential compression devices for deep vein thrombosis prophylaxis, a prescription of cephalexin for infection prophylaxis, and was advised to be non-weight bearing for six weeks post-op. 

Post-operative course

**Figure 3:**
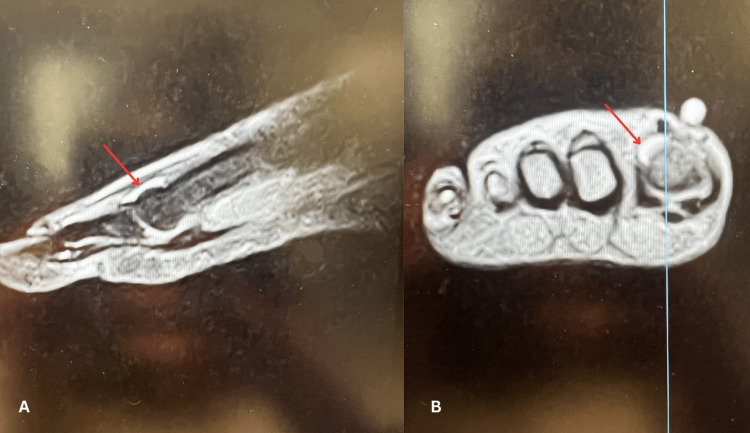
Static MRI sagittal (A) and coronal (B) images demonstrating tenosynovitis of the EHL tendon Tenosynovitis is indicated by the red arrows above. Plane of section in coronal image is shown by the light blue vertical line. EHL: Extensor hallucis longus

Follow-up at one week was notable for 9/10 pain but ultimately showed good progress with ability to move all digits with intact sensation. Radiographs indicated the surgical hardware and screws were in place with no evidence of failure or loosening. At the three-week follow-up, patient’s pain level had improved overall. However, ROM of the great toe was noted to be limited due to pain and swelling. Repeat radiographs revealed no changes to the surgical hardware and some callus formation noted in the fibula. The patient was recommended to maintain non-weight-bearing status and was placed in a boot. At six weeks, tenderness was noted over the EHL tendon and dorsiflexion of the great toe was rated at 2/5 strength. This weakness persisted at 10 weeks and an MRI was ordered, which revealed tendinosis with tenosynovitis in the EHL tendon with no other notable findings, shown below (Figures 3A, 3B). Physical therapy was continued.

At subsequent follow-up appointments, EHL strength and ROM remained diminished despite aggressive physical therapy, anti-spasmodics, and analgesics. Strength was rated at 1/5 with inability to dorsiflex the great toe past neutral. Other digits were noted to have 4/5 strength and ankle strength was rated as 5/5 for plantarflexion and dorsiflexion. Non-operative and operative measures were considered at this point, and tenolysis of the right EHL tendon was recommended and performed 28 weeks status post ORIF with IM nailing of the tibia.

One week following EHL tendon tenolysis, the patient reported very minimal pain with the absence of fever, chills, and drainage from surgical incision. On the physical exam, tenderness was noted over the right great toe and metatarsophalangeal joint with full plantar flexion and 5/5 strength. No active dorsiflexion of the right big toe was appreciated, and strength testing was deferred for later visits. By three weeks post tenolysis, mild active dorsiflexion and full passive flexion of the big toe was appreciated with only mild tenderness over the surgical incision. At this point, physical therapy to increase ROM and mobilization of the EHL was recommended.

Surgical techniques

ORIF With IM Nailing of the Tibia to Repair a Pilon Fracture Preformed One Week Post Injury

Fluoroscopic assistance was used to evaluate the status of the fracture. From there, a closed reduction was performed with a medial lateral screw being placed through the distal tibia plafond to rearticulate the tibiofibular joint. A second incision (2 cm) was made medial to the patella tendon. An awl and guide pin were used to open the proximal tibia, and a subsequent ball-tip guidewire was placed down through the tibia across the fracture site at the distal tibia. An IM nail and two lateral locking screws were then placed to stabilize the intra-articular fragments and cause fixation of distal fracture. A third incision (12 cm) was made laterally to expose the fibular fracture and a Biomet one-third tubular plate was placed. A 5 mL PRP injection was also given near the end of the procedure.

Tenolysis of the Right EHL Tendon Preformed Six Months Post Injury

A 5 cm incision was made over the distal third of the tibia over the EHL longitudinally. Remarkable scarring was noted upon exposure of the tendon. The initial incision was extended to 15 cm in response to this finding. A curette, Bovie, rongeur, and rasp were used to reconstruct the EHL to its normal course along the anterior portion of the tibia. Non-viable tissue was removed with Metzenbaum scissors and a scalpel. The extensor retinaculum was repaired with 2-0 Vicryl suture. The wound was irrigated with saline, hemostasis was restored with a Bovie cautery, and 5 mL of PRP was injected into the surgical sight. 

## Discussion

This case presents a rare complication of isolated EHL fibrosis following a traumatic pilon fracture in a 56-year-old male. Immediately following ORIF with IM nailing of the tibia, the patient’s strength in all digits was preserved. At his six-week follow up appointment, weakness and tenderness of the EHL tendon was noted. This persisted to 10 weeks post-op when an MRI demonstrated tendonitis and tenosynovitis of the EHL tendon. Conservative measures and physiotherapy failed, and the patient underwent tenolysis of the right EHL to repair function and mobility of the great toe. Three weeks following EHL tenolysis, the patient regained mild active dorsiflexion of the big toe with full passive flexion and no tenderness to palpation. 

Toe deformities secondary to ORIF of tibial shaft fractures are most commonly due to compartment syndrome or muscle adhesion to tendons [10]. This rare complication can be explained by either vascular compromise secondary to increased anterior compartment pressure or as a result of direct force from the fracture leading to muscle entrapment, adhesion, and eventual fibrosis [2]. Additionally, open surgical correction can lead to damaged blood supply and poor wound healing [10]. The anatomical location of the EHL on the distal fibula and interosseous membrane makes it susceptible to damage following fibular fracture and interosseous membrane damage, which may promote formation of a contracture [2]. Initial exam following surgery can be unremarkable, but the fibrosis may progress over the following weeks and the contracture may not be noted until several weeks post-op [10]. 

Known complications following pilon fracture repair include dehiscence, infection, varus malunion, non-union, joint stiffness, and post-traumatic arthritis [1]. To our knowledge, this is the only known case of EHL fibrosis following a pilon fracture. While this complication is rare, providers should be aware this complication may arise as a result of a pilon fracture especially in the context of concurrent distal fibular fracture. 

Throughout the care of this patient, frequent follow-up helped to aid in the diagnosis of EHL fibrosis. Ultimately MRI was needed to confirm the diagnosis. The patient was prescribed physical therapy, a home exercise regimen, and an anti-spasmodic (cyclobenzaprine) yet the EHL fibrosis persisted and ultimately tenolysis was required. The surgery was successful in correcting the deficit, and the patient was able to regain strength and ROM in his great toe. It is still unknown why EHL fibrosis occurs in some patients versus others. It is hypothesized that some patients may have a genetic predisposition to excess scar tissue production following injury, which may lead to fibrosis [8]. More research is needed to be done to understand why EHL fibrosis occurs and what can be done to prevent this complication following distal lower extremity fractures such as pilon fractures.

Preventative techniques, such as consistent and individualized physical therapy, are important in the discussion of limiting this complication even if it is unknown as to why it occurs. Stretching has been a constant point of discussion over whether it can limit contracture complications post injury. There appears to be not much clinically significant data that states stretching post injury is able to reduce the risk of contracture based on quantitative measurement of patient’s change in joint ROM when using stretching versus not during rehabilitation [8]. This study did not specifically investigate a post-trauma surgery population, however there is limited evidence to pull from regarding efficacy of stretching to prevent joint contracture.

Theoretically, stretching is not currently seen as an effective technique for maintaining full ROM of a joint, possibly due to the static nature of the movement. Mobility is often described as one of the most effective ways to preserve a joint. Usually physical therapy techniques will involve progressive dynamic movements that slowly rehabilitate the target joint back to its original ROM. Issues begin to present when one considers the importance of immobilization in the healing process of the injury post trauma. The balance between this and when to start mobility exercises will always be a challenge for rehabilitation plans. Additionally, pain management is another crucial factor that can limit a patient’s ability to perform certain exercises during rehabilitation. Other factors can include fear of injury, loss of confidence, loss of muscle strength, frailty, comorbidities, and various cognitive impairments [12]. It is essential that these factors be identified early in a patient’s recovery in order to facilitate a successful rehabilitation and avoid unwanted complications such as contracture. Other modalities of effective prevention of contracture are serial casting and neuromuscular electrical stimulation [6].

Another important consideration for prevention is the hardware and technique of the procedure. Specifically in the case of bone fractures, the placement of various anchoring modalities that help promote re-ossification can be part of the pathogenesis for contracture [2]. That is why it is important for physicians to consider the placement of foreign material in relation to the various tissue and neurovasculature present in the area when attempting to reduce a fracture intraoperatively.

## Conclusions

Our patient demonstrates the rare complication of EHL fibrosis following traumatic pilon fracture from a crush injury. An external fixator was not used in this patient's preoperative management. The patient received ORIF to repair the pilon fracture, followed by EHL tenolysis to regain mobility and function of the great toe. By three weeks post tenolysis, the patient regained mild active dorsiflexion of the great toe with full passive flexion. This case serves to provide better understanding of treatment and prognosis of the rare complication of EHL fibrosis following traumatic injury to the lower extremity. 
